# Coronary CT angiography for suspected acute coronary syndrome: sex-associated differences

**DOI:** 10.1007/s12471-021-01607-1

**Published:** 2021-08-06

**Authors:** M. Arslan, J. Schaap, A. Moelker, P. P. M. Rood, E. Boersma, K. Nieman, E. A. Dubois, A. Dedic

**Affiliations:** 1 Department of Cardiology, Erasmus University Medical Centre, Rotterdam, The Netherlands; 2 Department of Radiology and Nuclear Medicine, Erasmus University Medical Centre, Rotterdam, The Netherlands; 3 Department of Cardiology, Amphia Hospital, Breda, The Netherlands; 4 Department of Emergency Medicine, Erasmus University Medical Centre, Rotterdam, The Netherlands; 5 Departments of Cardiovascular Medicine and Radiology, Stanford University School of Medicine and Cardiovascular Institute, Stanford, CA USA; 6 Department of Intensive Care, Erasmus University Medical Centre, Rotterdam, The Netherlands

**Keywords:** Clinical trials, Acute coronary syndrome, Diagnostic testing, CT, Women

## Abstract

**Aim:**

The optimal diagnostic test in the work-up of suspected acute coronary syndrome (ACS) may differ between men and women. The aim of this study was to compare sex-associated differences between using a diagnostic strategy including early coronary computed tomography angiography (CCTA) and standard of care (SOC).

**Methods:**

In total, 500 patients who presented with symptoms suggestive of ACS at the emergency department were randomised between a diagnostic strategy supplemented with early CCTA and SOC.

**Results:**

Women were generally older than men (mean ± standard deviation 56 ± 10 vs 53 ± 10 years, *p* < 0.01) and were less often admitted to hospital (33% vs 44%, *p* = 0.02). Obstructive coronary artery disease on CCTA (> 50% luminal narrowing) was less frequently seen in women (14% vs 26%, *p* = 0.02), and ACS was diagnosed less often in women (5% vs 10%, *p* = 0.03). Women underwent less outpatient testing when early CCTA was used in the emergency department evaluation of suspected ACS (*p* = 0.008).

**Conclusion:**

Women had a lower incidence of obstructive CAD on CCTA and were less often admitted to hospital than men. They were subjected to less outpatient testing when early CCTA was used in the emergency department evaluation of suspected ACS.

**Supplementary Information:**

The online version of this article (10.1007/s12471-021-01607-1) contains supplementary material, which is available to authorized users.

## What’s new?

- In this study in patients with suspected acute coronary syndrome (ACS), women had a lower incidence of obstructive coronary artery disease on coronary computed tomography angiography (CCTA) than men.
- Women were less often admitted to hospital than men.
- Women underwent less outpatient testing when early CCTA was used in the emergency department evaluation of suspected ACS than men.

## Introduction

There are distinct pathophysiological differences in coronary artery disease (CAD) between men and women. Men are more likely to have obstructive epicardial CAD, while women are more prone to have coronary microvascular dysfunction [1]. In addition, disease perception may differ, both from the physician’s perspective—leading to sex-specific referral bias—and the patient’s own perception of chest discomfort, both of which can result in underrecognition of the burden of CAD in women [2]. The optimal diagnostic test in the work-up of suspected acute coronary syndrome (ACS) may therefore be different for men and women [3].

In this prespecified subanalysis of the Better Evaluation of Acute Chest Pain with Coronary Computed Tomography Angiography (BEACON) trial, we compared the clinical presentation, coronary computed tomography angiography (CCTA) results and the subsequent effect on downstream healthcare utilisation in men and women with suspected ACS.

## Methods

The study design, the criteria for enrolment and the primary results have been reported previously [4]. Briefly, in the BEACON trial, we randomised 500 patients suspected of having ACS (47% women, 79% of whom were evaluated with a high-sensitivity (hs) troponin assay) at the emergency department of seven hospitals to receive either standard of care (SOC) or a diagnostic strategy supplemented with early CCTA. In the SOC group, the attending physicians made clinical decisions regarding further testing, including repeated cardiac marker assessment, hospital admission, noninvasive tests and referral to invasive coronary angiography, according to relevant guidelines [5, 6]. The results of the main study showed that CCTA, which was applied early in the work-up of suspected ACS, is safe and is associated with less outpatient testing and lower costs. Data concerning particular endpoints, including coronary angiography, coronary revascularisation, hospital admission, length of stay, repeat emergency department visit and outpatient testing, were collected within 30 days of follow-up.

In the current analysis, non–sex-specific 99th percentile upper reference limits of normal were used for all troponin assays, conventional or high-sensitivity, with the exception of the Hs-TnI assay (Abbott ARCHITECT, Abbott Laboratories, Chicago, IL, USA), for which vendor-recommended sex-specific cut-off values were used. ACS was defined as either unstable angina pectoris or myocardial infarction (MI), according to the Third Universal Definition of MI [7].

### Statistical analysis

Continuous data are presented as mean ± standard deviation or median (interquartile range), and categorical data are presented as proportion (percentage). Differences between independent groups were compared using the analysis of variance or the Mann-Whitney U test for continuous variables, and the Fisher’s exact test or the Pearson’s chi-square test for categorical variables. All tests were two-tailed and a *p*-value < 0.05 was considered statistically significant. The study was conducted according to the principles of the Declaration of Helsinki and approved by the local institutional review boards. All patients provided written informed consent. The trial was registered at ClinicalTrials.gov (NCT01413282).

## Results

In the BEACON trial, women were generally older than men (56 ± 10 vs 53 ± 10 years, *p* < 0.01), less often active smokers and received less pharmacological treatment (Tab. 1). Table S1 in the Electronic Supplementary Material lists all troponin assays used, their characteristics and the algorithm with which they were implemented. ACS was less often diagnosed in women than in men (5% vs 10%, *p* = 0.03), and obstructive CAD on CCTA (> 50% luminal narrowing) was less frequently seen in women (14% vs 26%, *p* < 0.02).

**Table 1:** Baseline characteristics and study outcomes

Variable		Women (*n* = 236)	Men (*n* = 264)	*P*-value
Age, years		56 ± 10	53 ± 10	< 0.01
*Medication*				
Statin		43 (18.2)	73 (27.7)	0.01
Aspirin		35 (14.8)	48 (18.2)	0.32
Beta blocker		45 (19.1)	36 (13.6)	0.10
ACE inhibitor		23 (9.7)	35 (13.3)	0.22
Angiotensin receptor blocker		23 (9.7)	12 (4.5)	0.02
Calcium channel blocker		21 (8.9)	16 (6.1)	0.23
Diuretic agent		37 (15.7)	22 (8.3)	0.01
*Cardiovascular risk factors*				
Diabetes mellitus		25 (10.6)	39 (14.8)	0.16
Hypertension		102 (43.2)	119 (45.1)	0.68
Dyslipidaemia		76 (32.2)	101 (38.3)	0.15
Smoking (active)		65 (27.5)	106 (40.2)	< 0.01
Family history		101 (42.8)	109 (41.3)	0.73
GRACE score		85 ± 24	85 ± 25	0.96
*CCTA assessment of CAD*				0.02
Non-obstructive CAD	No stenosis	59 (48.4)	49 (39.2)	
	< 20% stenosis	13 (10.7)	12 (9.6)	
	20–49% stenosis	23 (18.9)	23 (18.4)	
Obstructive CAD	50–69% stenosis	13 (10.7)	23 (18.4)	
	> 70 stenosis	4 (3.3)	10 (8.0)	
Non-diagnostic		10 (8.2)	8 (6.4)	
*Outcomes*				
Coronary angiography		27 (11.4)	45 (17.0)	0.07
Coronary revascularisation		15 (6.4)	24 (9.1)	0.25
*ACS*		12 (5.1)	27 (10.2)	0.03
Unstable angina		5 (2.1)	6 (2.3)	0.91
Myocardial infarction		7 (3.0)	21 (8.0)	0.02
Hospital admission		77 (32.6)	110 (41.7)	0.04
Length of stay, hours		6.1 (4.5–15.0)	7.0 (4.7–24.5)	0.21
Repeat ED visit		17 (7.2)	15 (5.7)	0.49
Outpatient testing^a^		18 (7.6)	18 (6.8)	0.74
Baseline troponin level ≥ 99th percentile and non-obstructive CAD on CCTA		1 (0.4)	4 (1.5)	1.00

Values are mean ± standard deviation, *n* (%) or median (interquartile range)

*ACE* angiotensin-converting enzyme, *GRACE* Global Registry of Acute Coronary Events, *CCTA* coronary computed tomography angiography, *CAD* coronary artery disease, *ACS* acute coronary syndrome, *ED* emergency department

^a^ Outpatient testing consists of following cardiac tests: exercise electrocardiography, single-photon emission computed tomography, cardiac magnetic resonance imaging, coronary computed tomography angiography and invasive coronary angiography

Women were admitted less often (33% vs 42%, *p* = 0.04). The use of invasive coronary angiography (11% vs 17%, *p* = 0.07) and the rate of coronary revascularisation (6% vs 9%, *p* = 0.25) were not statistically different between women and men, although referral to invasive coronary angiography was numerically higher in men (Tab. 1).

Tab. 2 shows the study endpoints when comparing both diagnostic groups based on sex. Women underwent less outpatient testing when early CCTA was used in the emergency department evaluation of suspected ACS (*p* = 0.008).

**Table 2:** Study outcomes stratified by diagnostic group

Variable	SOC arm: women (*n* = 113); men (*n* = 137)	CCTA arm: women (*n* = 123); men (*n* = 127)	*P*-value
*Coronary angiography*			
Women	11 (9.7)	16 (13.0)	0.43
Men	20 (14.6)	25 (19.7)	0.26
*Coronary revascularisation*			
Women	6 (5.3)	9 (7.3)	0.53
Men	11 (8.0)	13 (10.2)	0.52
*ACS at discharge*			
Women	5 (4.4)	7 (5.7)	0.66
Men	12 (8.8)	15 (11.8)	0.41
*Unstable angina at discharge*			
Women	1 (0.9)	4 (3.3)	0.21
Men	2 (1.5)	4 (3.1)	0.36
*Myocardial infarction at discharge*			
Women	4 (3.5)	3 (2.4)	0.62
Men	10 (7.3)	11 (8.7)	0.68
*Hospital admission*			
Women	40 (35.4)	37 (30.1)	0.38
Men	64 (46.7)	49 (38.6)	0.33
*Length of stay*			
Women	6.0 (4.4–21.9)	6.3 (4.7–10.6)	0.58
Men	7.1 (4.5–27.1)	6.3 (4.8–17.4)	0.78
*Repeat ED visit*			
Women	11 (9.7)	6 (4.9)	0.15
Men	8 (5.8)	7 (5.5)	0.91
*Outpatient testing*^a^			
Women	14 (12.4)	4 (3.3)	0.008
Men	12 (8.8)	6 (4.8)	0.2

Values are *n* (%) or median (interquartile range)

*SOC* standard of care, *CCTA* coronary computed tomography angiography, *ACS* acute coronary syndrome, *ED* emergency department

^a^ Outpatient testing consists of following cardiac tests: exercise electrocardiography, single-photon emission computed tomography, cardiac magnetic resonance imaging, coronary computed tomography angiography and invasive coronary angiography

Tab. 3 shows the study endpoints when comparing both sexes within each diagnostic group. In the CCTA arm, men were more likely to be diagnosed with MI at discharge than women (*p* = 0.03). Furthermore, although not statistically significant, in the SOC arm, women were less likely to be admitted to hospital (*p* = 0.07) and had a shorter length of stay (*p* = 0.08) than men.

**Table 3:** Study outcomes stratified by sex

Variable	Women: SOC arm (*n* = 113); CCTA arm(*n* = 123)	Men: SOC arm (*n* = 137); CCTA arm (*n* = 127)	*P*-value
*Coronary angiography*			
SOC arm	11 (9.7)	20 (14.6)	0.25
CCTA arm	16 (13.0)	25 (19.8)	0.15
*Coronary revascularisation*			
SOC arm	6 (5.3)	11 (8.0)	0.40
CCTA arm	9 (7.3)	13 (10.3)	0.40
*ACS at discharge*			
SOC arm	5 (4.4)	12 (8.8)	0.18
CCTA arm	7 (5.7)	15 (11.8)	0.09
*Unstable angina at discharge*			
SOC arm	1 (0.9)	2 (1.5)	0.68
CCTA arm	4 (3.3)	4 (3.1)	0.96
*Myocardial infarction at discharge*			
SOC arm	4 (3.5)	10 (7.3)	0.20
CCTA arm	3 (2.4)	11 (8.7)	0.03
*Hospital admission*			
SOC arm	40 (35.4)	64 (46.7)	0.07
CCTA arm	37 (30.1)	49 (38.6)	0.16
*Length of stay*			
SOC arm	6.0 (4.4–21.9)	7.1 (4.5–27.1)	0.08
CCTA arm	6.3 (4.7–10.6)	6.3 (4.8–17.4)	0.70
*Repeat ED visit*			
SOC arm	11 (9.7)	8 (5.8)	0.25
CCTA arm	6 (4.9)	7 (5.5)	0.82
*Outpatient testing*^a^			
SOC arm	14 (12.4)	12 (8.8)	0.35
CCTA arm	4 (3.3)	6 (4.8)	0.54

Values are median (interquartile range) or *n* (%)

*SOC* standard of care, *CCTA* coronary computed tomography angiography,* ACS* acute coronary syndrome, *ED* emergency department

^a^ Outpatient testing consists of following cardiac tests: exercise electrocardiography, single-photon emission computed tomography, cardiac magnetic resonance imaging, coronary computed tomography angiography and invasive coronary angiography

## Discussion

In this prespecified analysis of the BEACON trial, women had a lower incidence of obstructive CAD on CCTA and were less often admitted to hospital than men. Women also underwent less outpatient testing when early CCTA was used in the emergency department evaluation of suspected ACS. Although not statistically significant, in the SOC arm, women were less likely to be admitted to hospital and had a shorter length of stay than men.

The presence of sex differences in the pathophysiology of ischaemic heart disease is becoming increasingly apparent [8, 9]. Previously, it has been shown that early CCTA may be a more efficient work-up for suspected ACS, especially in women. In a study by Truong et al., women showed a greater reduction in length of stay and hospital admission than men when early CCTA was used in the emergency department evaluation of suspected ACS [10]. The lower burden of CAD in women was thought to be a likely explanation for this difference, as physicians felt less need to perform downstream tests in patients with non-obstructive CAD on CCTA [10]. Although, we did not see similar reductions in hospital admission and length of stay, women underwent less outpatient testing than men when early CCTA was used. This reduction may also be due to the fact that women had a lower burden of CAD on CCTA, which in turn more often reassured physicians not to perform outpatient testing compared with men.

Although patients with angina without obstructive CAD have a better prognosis than those with obstructive epicardial CAD, they are still at higher risk of cardiovascular disease outcomes than the background population. Therefore, physicians should be vigilant in patients with recurring angina without obstructive epicardial CAD, especially in women, and initiate further evaluation of microvascular disease and treat accordingly.

A novelty in our study was the availability of hs-troponins for clinical decision-making in most of the patients. The introduction of hs-troponin assays has altered our perspective on MI and the way we practice medicine. These new cardiac biomarkers pick up myocardial injury fast and very precisely; serial low values almost certainly exclude MI [11, 12]. In the current study, the majority of the patients with available hs-troponins (> 90%) had normal levels (< 99th percentile of the upper limit of normal), which reassured treating physicians to discharge patients expeditiously, regardless of sex.

However, the improved sensitivity of these new biomarker assays has led to an increasing number of patients being diagnosed with myocardial injury *causa ignota*. Myocardial injury can be the result of type I MI associated with coronary plaque disruption or other types of MI, non-coronary heart disease or even non-cardiac diseases. Myocardial injury, irrespective of the cause, is associated with a less favourable prognosis and there are also sex-specific differences regarding the cause of injury [13–15]. Although this type of conclusions cannot be drawn from our own data, it is believed that women more often have other conditions than obstructive epicardial CAD that cause myocardial injury, such as coronary vasospasm or dissection [16, 17]. As a noninvasive anatomical modality, CCTA can serve as a gatekeeper of traditional coronary angiography by discriminating between obstructive and non-obstructive epicardial CAD.

### Study limitations

The current study does have some limitations that need to be highlighted. We have presented short-term data, but outpatient testing could also have taken place more than 30 days after the index presentation. In addition, the heterogeneity of troponin assays implemented in the current study could have impacted the diagnostic process and the downstream healthcare utilisation for both women and men. Finally, due to conflicting evidence and a lack of universally accepted sex-specific 99th percentile cut-offs, we were unable to implement sex-specific cut-offs for certain assays used in this study, with the exception of the hs-TnI assay (Abbott ARCHITECT).

## Conclusion

Compared with men, women had a lower incidence of obstructive CAD on CCTA, were less often admitted and underwent less outpatient testing when early CCTA was used in the emergency department evaluation of suspected ACS.

## Supplementary Information

**Tab. S1** Detailed information on the troponin assays used
