# Supplementary material for: Coronary CT angiography for suspected acute coronary syndrome: sex-associated differences
Source: Neth Heart J. 2021 Aug 6;29(10):518–24. doi: 10.1007/s12471-021-01607-1 (PMC8455783; doi:10.1007/s12471-021-01607-1)
Supplement: Supplementary file 1 — Tab. S1 Detailed information on the troponin assays used [file 12471_2021_1607_MOESM1_ESM.docx]

**Supplemental Table 1. Detailed information on the troponin assays used.**

| ***Assay*** | ***N (%)*** | ***Management*** | ***Interm. range***  ***(ng/L)*** | ***Level of detection (ng/L)*** | ***99^th^ percentile (ng/L)*** | ***10% Coeff. of variation (ng/L)*** |
| --- | --- | --- | --- | --- | --- | --- |
| ***hs-cTnT***  ***Roche Elecsys*** | *392 (78)* | *Serial measurement (3h interval).*  *Value above the pre-defined threshold or a significant rise is regarded as infarction* | *14-50* | *5* | *14* | *13* |
| ***TnT Gen 4***  ***Roche Elecsys*** | *87 (17)* | *Serial measurement (6h interval).*  *Value above the pre-defined threshold is regarded as infarction* | *10-30* | *10* | *10* | *30* |
| ***cTnI***  ***Abbott ARCHITECT*** | *13 (3)* | *Serial measurement (6h interval).*  *Value above the pre-defined threshold is regarded as infarction* | *28-50* | *10* | *28* | *32* |
| ***hs-cTnI ****  ***Abbott ARCHITECT*** | *4 (1)* | *Serial measurement (3h interval)*  *Value above the pre-defined threshold or a significant rise is regarded as infarction* | *16-34* | *1.2* | *Women:16 Men: 34* | *3* |
| ***AccuTnI***  ***Gen 3 enhanced Beckman Coulter*** | *4 (1)* | *Serial measurement (6h interval)*  *Value above the pre-defined threshold is regarded as infarction* | *40-60* | *10* | *40* | *60* |

* Vendor-recommended sex-specific 99^th^ percentile cut-offs were used for the hs-cTnI (Abbott ARCHITECT). Myocardial infarction was diagnosed according to the Universal Definition of Myocardial Infarction (1). Patients were diagnosed with MI in case of detection of a rise and/or fall of cardiac troponin values with at least one value above the 99^th^ percentile upper reference limit of normal and with clinical evidence of acute myocardial ischemia.

**References:**

1. Thygesen, K., et al., *Third universal definition of myocardial infarction.* J Am Coll Cardiol, 2012. **60**(16): p. 1581-98.
